# Paraoxonase-1 activity and AOPP levels in patients with type 2 diabetes mellitus

**DOI:** 10.5937/jomb0-53173

**Published:** 2026-01-28

**Authors:** Emre Hümeyra Oztürk, Nezaket Eren, Macit Koldas

**Affiliations:** 1 Cam and Sakura City Hospital, Department of Biochemistry, Istanbul, Turkey; 2 University of Yeni Yuzyil, Department of Biochemistry, Istanbul, Turkey; 3 Haseki Training and Research Hospital, Department of Biochemistry, Istanbul, Turkey

**Keywords:** AOPP, PON1, diabetes mellitus, HDL, complication, AOPP, PON1, dijabetes melitus, HDL, komplikacije

## Abstract

**Background:**

Paraoxonase 1 (PON1) is a calcium-dependent esterase and exerts antioxidant and antiatherogenic properties. Advanced oxidation protein products (AOPP) are a group of carbonylated protein products showing oxidant-mediated protein damage. This study aimed to determine serum PON1 activities and AOPP concentrations in diabetic patients and to evaluate these parameters in terms of their relationships with diabetes mellitus (DM) and related factors.

**Methods:**

A total of 93 patients diagnosed with type 2 DM and 30 healthy controls were enrolled in the study. Serum AOPP levels and PON1 activities were measured spectrophotometrically. Other biochemical parameters, including glucose, total cholesterol, HDL-C, LDL-C, triglycerides, HbA1c and clinical/demographic data, were measured in the routine blood chemistry laboratory and retrieved from patient files.

**Results:**

Serum PON1 activity was significantly lower in patients with DM (31.6 [21.49-48.45] U/mL) compared to controls (41.08 [29.07-54.35] U/mL) (p= 0.028). Serum AOPP concentration was significantly higher in diabetic patients (584.6 [453.8-778.6] pmol/L) than in controls (173.9 [98.77-224.1] pmol/L) (p&lt; 0.001). PON1 activity negatively correlated with AOPP concentration and positively with serum HDL-1 levels. AOPP concentration positively correlated with age, weight, HbA1c, glucose, total cholesterol, and LDL-C. A PON1 activity cut-off of ^25 U/mL predicted DM with a sensitivity of 36.56% and specificity of 90% (AUC: 0.634, p= 0.028). An AOPP concentration cut-off of &gt;340 mmol/L predicted DM with a sensitivity of 89.25% and specificity of 93.33% (AUC: 0.965). Both PON1 (OR: 10.821, 95% CI: 1.959-59.778, p= 0.006) and AOPP (OR: 190.068, 95% CI: 20.102-1797.148, p&lt; 0.001) were independently associated with DM after adjusting for age, sex, and weight.

**Conclusions:**

AOPP and PON1 may play a significant role in the development and progression of DM. In particular, serum AOPP concentrations appear to be distinctive among patients with new-onset DM.

## Abstract (sr)

**Uvod:**

Paraoksonaza 1 (PON1) je esteraza zavisna od kalcijuma koja ispoljava antioksidativna i antiaterogena svojstva. Napredni oksidacioni proteinski produkti (AOPP) predstavljaju grupu karbonilovanih proteinskih produkata koji ukazuju na oksidativno oštećenje proteina. Cilj ove studije bio je da se odrede aktivnosti PON1 i koncentracije AOPP u serumu kod pacijenata sa dijabetesom i da se ovi parametri procene u odnosu na dijabetes melitus (DM) i srodne faktore.

**Metode:**

U studiju je uključeno ukupno 93 pacijenta sa dijagnostifikovanim dijabetesom tipa 2 i 30 zdravih ispitanika u kontrolnoj grupi. Nivoi AOPP u serumu i aktivnosti PON1 su određeni spektrofotometrijski. Ostali biohemijski parametri, uključujući nivoe glukoze, ukupnog holesterola, HDL-C, LDL-C, triglicerida i HbA1c, kao i klinički/demografski podaci, određeni su u rutinskoj laboratoriji za biohemiju krvi i preuzeti iz dosijea pacijenata.

**Rezultati:**

Aktivnost PON1 u serumu je bila značajno niža kod pacijenata sa dijabetesom (31,6 [21,49-48,45] U/mL) u poređenju sa kontrolnom grupom (41,08 [29,07-54,35] U/mL) (p= 0,028). Koncentracija AOPP u serumu je bila značajno viša kod pacijenata sa dijabetesom (584,6 [453,8-778,6] pmol/L) u odnosu na kontrolnu grupu (173,9 [98,77-224,1] pmol/L) (p&lt; 0,001). Aktivnost PON1 je bila u negativnoj korelaciji sa koncentracijom AOPP i pozitivnoj sa nivoima HDL-1 u serumu. Koncentracija AOPP pozitivno je korelisala sa godinama starosti, telesnom težinom, HbA1c, glukozom, ukupnim holesterolom i LDL-C. Prag aktivnosti PON1 od ^25 U/mL predvideo je DM sa osetljivošću od 36,56% i specifičnošću od 90% (AUC: 0,634, p= 0,028). Prag koncentracije AOPP od &gt;340 mmol/L predvideo je DM sa osetljivošću od 89,25% i specifičnošću od 93,33% (AUC: 0,965). I PON1 (OR: 10,821, 95% CI: 1,959-59,778, p= 0,006) i AOPP (OR: 190,068, 95% CI: 20,102-1797,148, p&lt;0,001) bili su nezavisno povezani sa DM nakon prilagođavanja za godine, pol i telesnu težinu.

**Zaključak:**

AOPP i PON1 mogu da igraju značajnu ulogu u razvoju i progresiji DM. Posebno se koncentracije AOPP u serumu ističu kao diferencijalni marker kod pacijenata sa novodijagnostifikovanim dijabetesom.

## Introduction

Diabetes mellitus (DM), in addition to its impact on blood glucose and insulin levels, leads to metabolic dysfunction, lipid and protein metabolism disorders, oxidative stress (OS) and accelerated arteriosclerosis [1] [2]. It is also associated with ocular, neurological, cardiovascular and urogenital manifestations [3]. The burden and costs of DM regarding prevalence and number of patients with complications have increased dramatically, especially in low- and middle-income countries [4]. The global prevalence was approximately 10.5% in 2021 [5] and is foreseen to accelerate swiftly by 2045, largely due to ageing populations and sedentary lifestyles [6]. The pathogenesis of diabetic complications is multifactorial and uncertain; a number of hyperglycemia-related pathways, such as OS and inflammation, have been implicated in the development and progression of these conditions [7]. An in-depth understanding of hyperglycemia-induced OS is required to characterise the complete pathogenesis of adverse outcomes associated with DM.

Paraoxonase 1 (PON1) is a calcium-dependent esterase produced primarily in the liver, which is then secreted into the circulation in conjunction with high-density lipoprotein particles (HDL) [8] [9]. Although HDL binds many proteins that have functional roles, the anti-oxidative and antiatherogenic properties of HDL are mainly attributed to PON1 [10]. Alterations in PON1 activity have been associated with many disorders, including atherosclerosis, obesity, cancer, kidney diseases, and DM [8]. Although previous studies have shown decreased PON1 levels in patients with DM, the underlying mechanisms are still poorly understood [11].

Advanced oxidation protein products (AOPPs) are a group of carbonylated protein products generated by the reaction of plasma proteins and chlorinated oxidants, including hypochlorous acid and chloramine. They are well-recognised as being oxidant-mediated markers of protein damage [12] [13]. Higher AOPP levels have been detected in patients with cardiovascular diseases, hypertension, atherosclerosis, osteoporosis and neuroinflammatory diseases [13] [14]. Although some studies have shown that AOPP levels are associated with DM and the presence or severity of DM complications, the molecular mechanisms of these changes and associations with other OS markers are unclear [15].

Our aim was to determine serum levels of AOPP and PON1 in patients diagnosed with DM, to assess diagnostic performance, and to determine whether AOPP and PON1 levels were associated with other DM-related factors in our study population.

## Materials and methods

### Patients and study design

The present research was designed as a single-centre cross-sectional study and was conducted at Haseki Training and Research Hospital, Istanbul, Turkey. A total of 93 patients diagnosed with type 2 DM and 30 healthy controls were enrolled in the study. The diagnosis of type 2 DM was based on the criteria of the World Health Organization: A fasting plasma glucose level of ≥7.0 mmol/L or a 2-hour postprandial plasma glucose level of ≥11.1 mmol/L [16]. The control group consisted of 30 subjects who were not suffering from DM and had no history of coronary artery disease, metabolic syndrome, or liver, kidney and thyroid diseases. Participants with acute or chronic infections, malignancy, chronic inflammatory conditions, medical conditions that could affect OS levels, alcohol consumption, substance abuse, and those within pregnancy or within 1 year postpartum were excluded from the study. None of the healthy controls had been receiving lipid-lowering therapy (drug usage or physical activity), antioxidant treatment, or were keeping any specific diet. Demographics and other information, including age, sex, weight and height, concomitant disorders, and current medications, were obtained from patient files.

All research procedures were evaluated and accepted by the Research Ethics Committee of Haseki Training and Research Hospital and were conducted in agreement with the ethical standards specified in the Declaration of Helsinki. Written and verbal informed consent was obtained from all participants prior to their participation in this study.

### Biochemical analysis

After overnight fasting, blood samples were drawn from the antecubital vein and were centrifuged at 1300 g for 15 minutes to separate serum. Aliquoted samples were kept at -20°C and thawed immediately before biochemical analysis. Serum glucose, total cholesterol, triglyceride, HDL-cholesterol (HDL-C), urea and creatinine were measured with photometric methods on a Roche Modular P800 analyser using commercial kits (Roche Diagnostics, Mannheim, Germany). Glycated haemoglobin (HbA1c) levels were determined using a high-performance liquid chromatography (HPLC) method on the TOSOH G7 analyser (TOSOh Europe NV, Tessenderlo, Belgium). Serum low-density lipoprotein cholesterol (LDL-C) levels were calculated using the Friedewald formula. For triglyceride levels >22.2 mmol/L, LDL-C was measured directly using direct homogeneous assay.

PON1 activity was measured by modifying the method developed by Eckerson [17]. The method was based on the conversion of phenyl acetate to *p*-nitrophenol and acetate by paraoxonase found in human serum. Butyryl choline was added to the reaction mixture to inhibit esterases. The increase in the absorbance of the formed *p*-nitrophenol was measured spectrophotometrically at a wavelength of 270 nm using a UV-visible spectrophotometer (Schimadzu UV 1601, Kyoto, Japan). One unit of PON1 activity was defined as the enzyme activity that produces 1 mmol of *p*-nitrophenol per minute, and results were reported in U/mL.

Spectrophotometric measurement of AOPP was performed according to the method described by Witko-Tarsat et al. [12]. Briefly, 160 μL of phosphate-buffered saline (PBS) reagent was added to 10 μL of serum and incubated for 25 seconds. Then, 20 μL acetic acid and 10 μL of 1.16 mol/L potassium iodide solution were added to each well, respectively, and incubated. The absorbance was read at 340 nm. Chloramine T solution was used as a calibrator, and chloramine T absorbance at 340 nm was observed to be linear in the range of 0-100 μmol/L. AOPP concentrations were expressed in chloramine equivalents (μmol/L).

### Statistical analysis

Statistical analyses were tested for a significance threshold of <0.05 (*p*-value). The IBM SPSS for Windows software, Version 25.0 (IBM Corp., Armonk, NY, USA) was used for analyses. The normality of distribution was tested with histograms and Q-Q plots. Descriptive statistics included mean±standard deviation for normally distributed continuous variables, median (25th percentile-75th percentile) for nonnormally distributed continuous variables, and frequency (percentage) for categorical variables. Between-group analysis of continuous variables was performed using the Student's t-test or Mann-Whitney *U* test, depending on the normality of distribution. Between-group analysis of categorical variables was performed by using the chi-square test with continuity correction. Spearman correlation coefficients were calculated to evaluate directional relationships between numerical variables. Prediction performances of the PON1 and AOPP variables were assessed by using receiver operating characteristic (ROC) curve analysis. Optimal cut-off points were determined with the Youden index. Unadjusted and adjusted odds ratios were calculated via univariable and multivariable logistic regression analysis.

## Results

**Figure 1:**
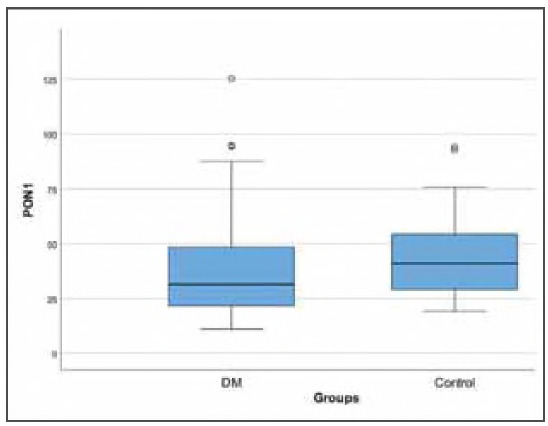
Box plot of the PON1 with regard to groups.

**Figure 2:**
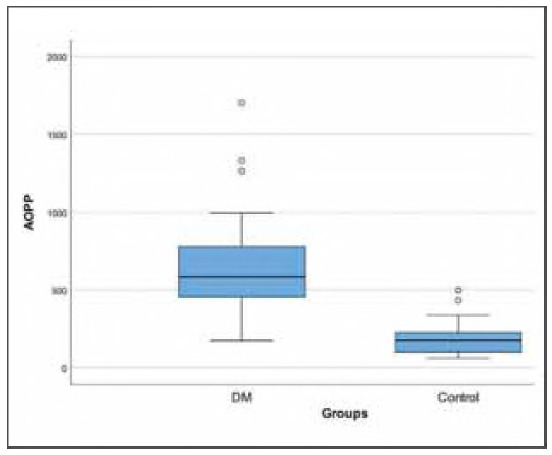
Box plot of the AOPP with regard to groups.

A total of 93 diabetic patients and 30 healthy subjects were enrolled in the study. The median age of the diabetic patients was 56 years (45-65), whereas the control group had a median age of 31 years (25-42), with a statistically significant difference (p<0.001). Sex distribution was similar (*p* = 0.077). The patient group had higher weight values (73.26± 15.33 kg) compared to controls (67.07±10.33 kg) (*p* = 0.014). Diabetic patients had higher glucose, HbA1c, total cholesterol, LDL-C and creatinine levels compared to controls (all, *p*<0.05). Serum PON1 activity was 31.6 (21.49-48.45) U/mL in patients with DM and 41.08 (29.07-54.35) U/mL in controls (*p* = 0.028) (Figure 1). Serum AOPP concentration was significantly higher in patients with DM [584.6 (453.8-778.6) μmol/L] than in controls [173.9 (98.77-224.1) μmol/L] (p<0.001) (Figure 2). The demographic and biochemical features of participants are summarised in Table 1.

**Table 1:** Demographics and biochemical characteristics with regard to groups. HbA1c: Glycated haemoglobin, HDL-C: High-density lipoprotein-cholesterol, LDL-C: Low-density lipoprotein-cholesterol, PON1: Paraoxonase 1, AOPP: Advanced oxidised protein products. Descriptive statistics were presented by using mean±standard deviation for normally distributed continuous variables, median (25th percentile - 75th percentile) for non-normally distributed continuous variables and frequency (percentage) for categorical variables. † Student's t-test, ‡ Mann Whitney U test, § Chi-square test

Variables	Groups		*P*
	Diabetes mellitus (n=93)	Control (n=30)	
Age, years	56 (45-65)	31 (25-42)	<0.001^‡^
Sex			
Female, n	65 (69.89%)	15 (50.00%)	0.077^§^
Male, n	28 (30.11%)	15 (50.00%)	
Weight, kg	73.26±15.33	67.07±10.33	0.014^‡^
HbA1c, mmol/mol	60.6 (47.6 - 78.1)	34.4 (29.0-43.2)	<0.001^‡^
Glucose, mmol/L	7.65 (5.88-11.88)	4.77 (4.50-5.55)	<0.001^‡^
Cholesterol, mmol/L	5.23±1.32	4.36±0.78	<0.001^†^
Triglycerides, mmol/L	1.75±1.19	1.44±0.35	0.137^†^
HDL-C, mmol/L	1.20±0.29	1.24±0.25	0.478^†^
LDL-C, mmol/L	3.14±1.18	2.46±0.85	0.003^†^
Urea, mmol/L	5.64 (4.32-7.14)	4.73 (4.15-5.64)	0.097^‡^
Creatinine, μmol/L	79.6 (70.7-97.2)	66.3 (61.9-79.6)	0.001^‡^
PON1 activity, U/mL	31.60 (21.49-48.45)	41.08 (29.07-54.35)	0.028^‡^
AOPP activity, μmol/L	584.6 (453.8-778.6)	173.9 (98.77-224.1)	<0.001^‡^

The results of correlation analyses are presented in Table 2. PON1 activity was negatively correlated with AOPP concentration (*r*=-0.270 and *p* = 0.003). PON1 activity was also correlated with serum HDL-C in a positive manner and with serum creatinine levels in a negative manner (*p* = 0.003 and 0.036, respectively). AOPP concentration was positively correlated with age, weight, HbA1c, glucose, total cholesterol and LDL-C (all, *p*<0.005).

**Table 2:** Correlation analysis between PON1, AOPP and other variables. HbA1c: Glycated haemoglobin, HDL-C: High-density lipoprotein-cholesterol, LDL-C: Low-density lipoprotein-cholesterol, PON1: Paraoxonase 1, AOPP: Advanced oxidised protein products. *r*: Spearman correlation coefficient.

Variables		PON1 activity	AOPP activity
Age	*r*	-0.045	0.354
	*p*	0.624	<0.001
Sex, Male	*r*	-0.010	-0.146
	*p*	0.910	0.107
Weight, kg	*r*	0.056	0.237
	*p*	0.537	0.008
HbA1c, mmol/mol	*r*	-0.074	0.487
	*p*	0.414	<0.001
Glucose, mmol/L	*r*	-0.106	0.423
	*p*	0.244	<0.001
Total cholesterol, mmol/L	*r*	0.127	0.299
	*p*	0.162	0.001
Triglycerides, mmol/L	*r*	0.209	0.061
HDL-C, mmol/L	*p*	0.089	0.625
	*p*	0.003	0.200
LDL-C, mmol/L	*r*	0.010	0.327
	*p*	0.915	<0.001
Urea, mmol/L	*r*	-0.097	0.068
	*p*	0.324	0.488
Creatinine, μmol/L	*r*	-0.205	0.190
	*p*	0.036	0.053
PON1 activity, U/mL	*r*	-	-0.270
	*p*	-	0.003

**Figure 3:**
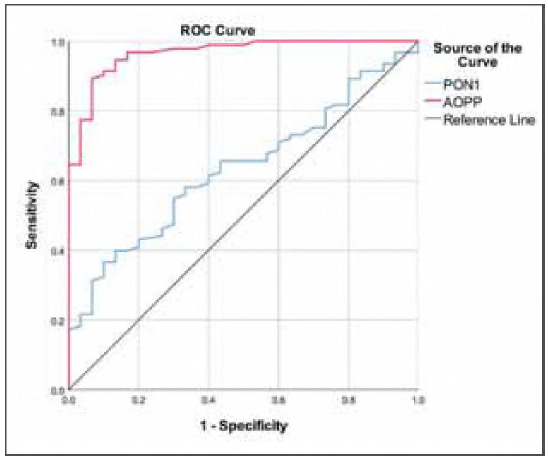
ROC curves of the PON1 and AOPP to predict diabetes mellitus.

The receiver operating characteristics (ROC) curve analysis was performed to obtain the optimal cut-off value of PON1 activity and AOPP concentrations for the diagnosis of DM (Table 3) (Figure 3). ≤25 U/mL of PON1 activity as the cut-off point for prediction of DM revealed a sensitivity and specificity of 36.56% and 90%, respectively (Area under the curve (AUC): 0.634, and *p* = 0.028). The optimum cut-off value of >340 μmol/L for AOPP concentration showed a sensitivity of 89.25% and specificity of 93.33% for diagnosing DM, with an AuC of 0.965. Multiple logistic regression analysis revealed that PON1 (OR: 10.821, 95% CI: 1.959-59.778, *p* = 0.006) and AOPP (OR: 190.068, 95% CI: 20.102-1797.148, *p*<0.001) were independently associated with DM presence after adjusting for age, sex and weight.

**Table 3:** Performance of PON1 and AOPP to predict diabetes mellitus by ROC curve analysis. PON1: Paraoxonase 1, AOPP: Advanced oxidised protein products. ROC: Receiver operating characteristic, PPV: Positive predictive value, NPV: Negative predictive value, AUC: Area under ROC curve, CI: Confidence interval, OR: Odds ratio. (1) Adjusted by age, sex and weight.

Variables	PON1 activity	AOPP activity
Cut-off	≤25	>340
Sensitivity	36.56%	89.25%
Specificity	90.00%	93.33%
Accuracy	49.59%	90.24%
PPV	91.89%	97.65%
NPV	31.40%	73.68%
AUC (95% CI)	0.634 (0.531-0.737)	0.965 (0.934-0.997)
p for AUC	0.028	<0.001
OR (95% CI)	5.186 (1.463-18.381)	116.200 (23.995-562.710)
p for OR	0.011	<0.001
Adjusted OR (95% CI) ^(1)^	10.821 (1.959-59.778)	190.068 (20.102-1797.148)
p for adjusted OR	0.006	<0.001

## Discussion

This study aimed to determine serum PON1 activities and serum AOPP concentrations in patients diagnosed with DM and to evaluate the associations of these parameters with DM and DM-related features. We demonstrated significantly higher AOPP concentrations and lower PON1 activities among diabetic patients compared to healthy individuals. A negative correlation was observed between PON1 activity and AOPP concentration in diabetic patients. Cut-off point values of ≤25 U/mL for PON1 activity and >340 μmol/L for AOPP concentration showed high sensitivity and specificity for DM detection. Patients with high AOPP and low PON1 were more likely to have DM than controls after adjusting for other factors.

Under different metabolic dysfunctions, including DM, the oxidative balance may be disrupted and can elevate OS, which is a well-known contributor to the progression of many disorders, including cancer, neurodegenerative and inflammatory disorders, and cardiovascular disease [7]. DM is a silent, progressive and chronic metabolic condition with a complicated pathogenesis in which both genetic and environmental determinants promote its development [1]. The significance of OS in the pathogenesis of DM and its micro- and macrovascular complications has been supported by comprehensive evidence [18]. Diabetic patients often have increased OS, characterised by a depleted antioxidant system as well as high levels of glycosylation and peroxidation. Persistent hyperglycemia can lead to the formation of advanced glycation end products (AGEs) and the activation of protein kinase C and NADPH oxidase, eventually resulting in the overproduction of reactive oxygen species (ROS). The imbalance in oxidative and antioxidative parameters contributes to the development and progression of insulin resistance (IR) and DM [18] [19]. Non-enzymatic glycation of macromolecules such as lipids, proteins, and DNA leads to structural and functional changes, contributing to OS [20] [21] and various diabetic complications [22]. In addition, intracellular antioxidant capacity is reduced through various mechanisms which activate cytokines/chemokines, pro-inflammatory mediators and growth factors, worsening the OS-favoring microenvironment and leading to tissue injury and inflammation [23]. Due to the structural similarity between AOPPs and AGEs, AOPPs can cause biological impacts similar to AGEs, such as the induction of pro-inflammatory cytokines and adhesive molecules, which creates a self-sustaining cycle of excessive ROS generation and worsening oxidative damage to proteins [24]. Studies have reported that AGEs and AOPP may contribute to DM and its complications and that their concentrations may be associated with the presence or severity of insulin resistance and diabetic complications. Therefore, we aimed to assess the AOPP level together with the activity of PON1 in diabetic patients without complications and compared them with those of healthy subjects. Consistent with the literature, we found increased AOPP levels in patients with DM. We also showed positive relationships between AOPP and age, weight, glucose, HbA1c, total cholesterol, and LDL-C. Similar to our results, Heidari et al. demonstrated that serum AOPP level was higher in newly diagnosed diabetics than in controls and was further increased among patients with longer diabetes duration (1-5 years) compared to the new-onset subgroup [15]. Ero lu et al. [25] reported an increase in serum AOPP levels in patients with type 2 DM but did not find an association with the presence of diabetic neuropathy. Liang et al. showed that plasma AOPP levels were higher in normoalbuminuric patients with type 2 DM and that increased AOPP level was an independent risk factor for endothelial dysfunction [26]. Animal models with induced DM also support these findings. An experimental streptozocin-induced diabetic rat model showed increased AOPP levels and decreased PON1 activities at baseline, and co-administration of lysine, vitamin C and zinc to rats resulted in decreased blood and protein glycation (reduced AGEs) and improved insulin secretion, lipid profile, HDL functionality, antioxidant status, and kidney function (increased LCAT and PON1, decreased FRAP, AOPP, urea and creatinine) [27]. Our data indicate that AOPP could be contributing to the pathogenesis of DM and that a >340-μmol/L threshold of AOPP can distinguish patients with DM from those without. Correlations between AOPP and serum glucose and HbA1c levels indicate that poor glycemic control presents increased levels of AOPP that may cause oxidative protein damage and inflammation. The positive relationship between AOPP and parameters of serum lipid profile supports the notion that AOPP is associated with occurrence and progression of diabetic vascular complications. The relationships shown in this study should be examined in research involving larger sample sizes and the analyses must be extended to patients with DM complications.

In recent years, PON1 has also gained interest for its role in the hydrolysis of lipolactones, which are components of damaged oxidised lipoproteins [28]. PON1 has been shown to reduce LDL oxidation, prevent the accumulation of oxidised LDL by increasing cholesterol efflux, and may inhibit atherosclerosis at an early stage by terminating the pro-inflammatory response triggered by oxidised LDL and other oxidised derivatives of different compounds [11] [29]. Indeed, PON1 activity has been reported to be inversely associated with the risk of cardiovascular diseases and DM [10] [11]. Low PON1 activity in diabetic patients is believed to result from a reduction of its specific activity through non-enzymatic glycation, which is a hallmark of hyperglycemia [30]. Therefore, one of the most important factors leading to the creation of dysfunctional HDL could be decreased PON1 activity in diabetic patients, leading to acceleration of atherosclerosis and, thus, cardiac complications and adverse cardiac outcomes [31]. In addition, the role of hyperglycemia in directly promoting OS by depleting natural antioxidants and inducing the overproduction of ROS makes diabetic patients more sensitive to the loss of PON 1-induced protective properties, potentially exposing LDL particles to considerably elevated levels of peroxidation [32]. Consistent with the literature in this field, we found decreased PON1 activity in patients with DM compared to the healthy controls. We also demonstrated an inverse relationship between AOPP levels and PON1 activities. Our results support the hypothesis that inhibition of lipid oxidation may be reduced in DM patients due to low PON1 enzyme activity and that PON1 plays a critical role in the relationship between OS and hyperglycemia. The negative correlation between PON1 and AOPP indicates that both parameters may contribute to the pathogenesis of DM and its vascular complications. However, since this was a cross-sectional study, the temporal relationships between these effects are unclear and further research is necessary to understand preceding or predisposing factors.

## Conclusion

In conclusion, our study demonstrated that AOPP and PON1 may play a significant role in the development and progression of DM and that serum AOPP concentrations and PON1 activities can distinguish patients with DM from those without. In this context, AOPP concentrations appear to have very high accuracy in detecting DM. Although many of the correlations detected in this study were weak to moderate, we believe the associations of PON1 and AOPP with other factors indicate that advanced research is warranted to understand how these parameters tie in to DM pathophysiology. Further studies with larger sample sizes are needed to confirm the effect of AOPP and PON1 on insulin metabolism and other DM-related properties.

## Dodatak

### Conflict of interest statement

All the authors declare that they have no conflict of interest in this work.
